# The origin and distribution of the main oxygen sensing mechanism across metazoans

**DOI:** 10.3389/fphys.2022.977391

**Published:** 2022-10-17

**Authors:** Bing Song, Luca David Modjewski, Nils Kapust, Itzhak Mizrahi, William F. Martin

**Affiliations:** ^1^ Department of Biology, Institute for Molecular Evolution, Heinrich-Heine-Universität Düsseldorf, Düsseldorf, Germany; ^2^ Department of Life Sciences, Ben-Gurion University of the Negev and the National Institute for Biotechnology in the Negev, Marcus Family Campus, Be’er-Sheva, Israel

**Keywords:** anaerobes, metazoan, origin, oxygen sensing mechanism, terminal oxidase

## Abstract

Oxygen sensing mechanisms are essential for metazoans, their origin and evolution in the context of oxygen in Earth history are of interest. To trace the evolution of a main oxygen sensing mechanism among metazoans, the hypoxia induced factor, HIF, we investigated the phylogenetic distribution and phylogeny of 11 of its components across 566 eukaryote genomes. The HIF based oxygen sensing machinery in eukaryotes can be traced as far back as 800 million years (Ma) ago, likely to the last metazoan common ancestor (LMCA), and arose at a time when the atmospheric oxygen content corresponded roughly to the Pasteur point, or roughly 1% of present atmospheric level (PAL). By the time of the Cambrian explosion (541–485 Ma) as oxygen levels started to approach those of the modern atmosphere, the HIF system with its key components HIF1α, HIF1β, PHD1, PHD4, FIH and VHL was well established across metazoan lineages. HIF1α is more widely distributed and therefore may have evolved earlier than HIF2α and HIF3α, and HIF1β and is more widely distributed than HIF2β in invertebrates. PHD1, PHD4, FIH, and VHL appear in all 13 metazoan phyla. The O_2_ consuming enzymes of the pathway, PHDs and FIH, have a lower substrate affinity, K_m_, for O_2_ than terminal oxidases in the mitochondrial respiratory chain, in line with their function as an environmental signal to switch to anaerobic energy metabolic pathways. The ancient HIF system has been conserved and widespread during the period when metazoans evolved and diversified together with O_2_ during Earth history.

## Introduction

**FIGURE 1:**
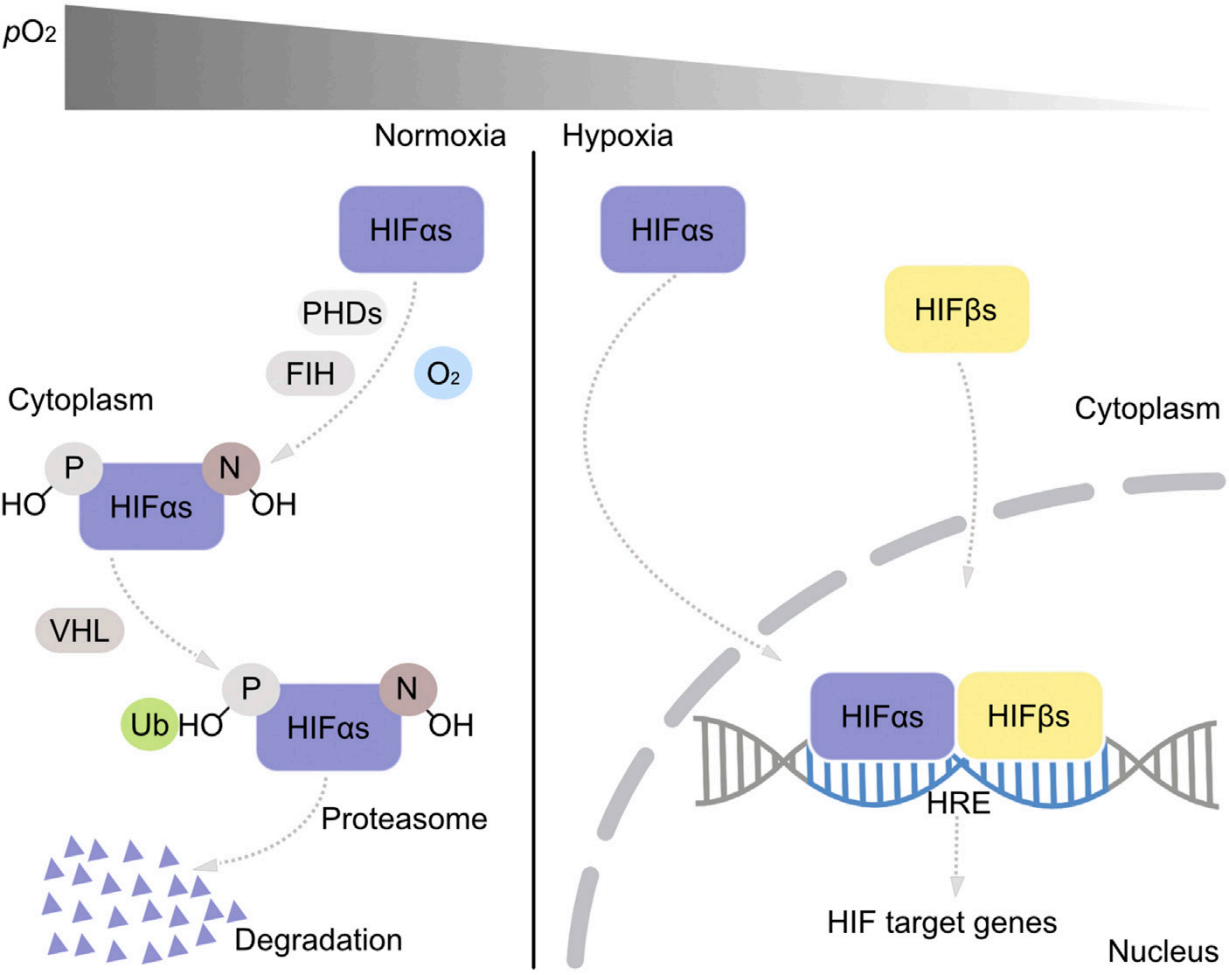
The HIF oxygen sensing pathway. Under normoxic conditions, proline (P) and asparaginyl (N) residues in HIFαs are first recognized and degraded by activated prolyl hydroxylases (PHDs) and asparaginyl hydroxylase (FIH). The hydroxylated proline residues (P-OH) bind on HIFαs and are ubiquitinated by the VHL and degraded by proteasomes within the cytoplasm. Under hypoxia, PHDs and FIH are inactivated and HIFαs are transported into the nucleus and form heterodimers with HIFβs. This heterodimer binds on the hypoxia responsive element (HRE) in the DNA structure and functions as a transcription factor activating or repressing the transcription activities of the downstream genes.

Oxygen on Earth stems from cyanobacterial photosynthesis. During Earth history, there were two main phases of atmospheric oxygen content change (Mills et al., 2022). The first was the great oxidation event, GOE, around 2.4 billion years (Ga) ago (Bekker et al., 2004), followed by almost 2 Ga of low oxygen levels and a second, late rise of oxygen corresponding to the origin of land plants and animals about 500 million years (Ma) ago (Lyons et al., 2014). The metazoan lineage arose roughly 700–1000 Ma ago, long before the origin of land plants, during a phase of Earth history when oxygen levels were much lower than today’s. The first metazoans were thus well adapted to low oxygen environments from the outset of their evolution, but adapted to rising oxygen levels during evolution, particularly with the transition to life on land in a highly oxic atmosphere starting about 450 Ma ago (Martin et al., 2021). Oxygen sensing pathways in animals are integral to their evolution during changing oxygen concentrations (Hammarlund et al., 2020) in Earth history. The main oxygen sensing pathway in animals is mediated by hypoxia-inducible factors, HIFs (Semenza 2001; Kaelin and Ratcliffe 2008; Zhang et al., 2014; Ivan and Kaelin 2017). The alpha subunit of HIF1, HIF1α is posttranslational modified by prolyl hydroxylases (PHD1—4), which catalyze the O_2_ dependent hydroxylation of prolyl residues, inducing ubiquitinylation of HIF by the von Hippel Lindau tumor suppressor (VHL) (Brihimi-Horn and Pouysségur 2009), leading to HIF1α degradation by proteasomes (Figure 1). The factor inhibiting HIF, FIH, hydroxylates asparaginyl residues in the alpha subunit of HIFs (HIF1—3α), in an O_2_ dependent reaction, which inhibits proline hydroxylation (Figure 1). HIF1α and PHDs are constitutively expressed. When O_2_ is lacking, the alpha subunit of HIFs is not hydroxylated by PHDs and therefore not degraded, but accumulates and is transported into the cell nucleus to create a heterodimer with the beta subunit of HIFs (HIF1β and 2β). The HIFαβ-heterodimer acts as the transcription factor, which binds to corresponding hypoxia response elements (HRE) of the promoters of the HIF-target genes to stimulate or repress the downstream gene transcription activities which can elicit a series of biological responses (Brihimi-Horn and Pouysségur 2009) (Figure 1). As it relates to energy metabolism, the main biological response governed by the HIF pathway is a shift from aerobic energy metabolism to anaerobic energy metabolism, which in land mammals, diverts pyruvate flux away from mitochondria and O_2_ dependent terminal oxidases towards cytosolic fermentations (Samanta and Semenza 2018).

The mechanism of the main oxygen sensing pathway is well known and its origin and distribution have also been widely studied from the phylogenetic perspective (Rytkönen et al., 2011; Rytkönen and Storz 2011; Mills et al., 2018; Rytkönen 2018; Graham and Barreto 2019; Graham and Barreto 2020) but less so from the geological perspective (Taylor and McElwain 2010; Hammarlund 2020). One hypothesis is that the main oxygen sensing pathway may have already occurred in the common ancestor of metazoans (Hammarlund et al., 2018; Hammarlund 2020). PHD like prolyl hydroxylases have been reported in unicellular eukaryotes such as *Dictyostelium* (Van der Wel et al., 2005) and *Schizosaccharomyces pombe* (Lee et al., 2009) and pathogenic proteobacteria like *Vibrio cholerae* (Aravind and Koonin 2001; McDonough et al., 2006) and *Pseudomonas aeruginosa* (Aravind and Koonin 2001; Scotti et al., 2014). The HIFα/PHD/VHL pathway is conserved in all animals but is not found in choanoflagellates (*Monosiga brevicollis*) or other protists (Loenarz et al., 2011), it is present in all eumetazoans, except *Ctenophera* (Mills et al., 2018). A recent study showed that choanoflagellates have PHD but not HIF and VHL (Rytkönen 2018). HIF1α is conserved among most metazoans while HIF2α appeared later (Graham and Presnell 2017). Some studies have suggested that the HIF pathway was lacking in the last common ancestor of animals and is not ubiquitous across metazoans (Mills et al., 2018; Graham and Barreto 2020). From the geological perspective, the HIF system requires the presence of O_2_, with HIF1α, HIF1β, PHD2, and VHL being the oldest components, HIF2α and PHD3 were suggested to have appeared around 460 to 421 Ma ago when the atmospheric oxygen level was lower than the present, whereby PHD1 and HIF3α are thought to have arisen most recently (about 312 Ma ago) with contemporary atmospheric oxygen levels (Taylor and McElwain 2010). FIH1 is missing in fruit flies and nematodes but not in intermediate beetles (Taylor and McElwain 2010).

Here, we focus on the origin and distribution of the HIF/PHD/FIH/VHL oxygen sensing pathway within metazoans from the perspective of oxygen in Earth history, addressing the two phases of the appearance of its essential components: First, the presence of HIF/PHD/FIH/VHL pathway related genes—HIFαs, HIFβs, PHDs, FIH, and VHL—across metazoan and non-metazoan phyla, and the oxygen affinity of oxygen sensing enzymes (PHDs and FIH) were compared to terminal oxidases in mitochondria by the measure of substrate affinity for O_2_, K_m_^app^ (O_2_).

## Methods

### Identification and presence of hypoxia induced factor related genes

From the National Center for Biotechnology Information (NCBI) (Agarwala et al., 2018), Kyoto Encyclopedia of Genes and Genomes (KEGG) (Kanehisa et al., 2016), European Molecular Biology Laboratory (EMBL) (Nightingale et al., 2017), and Universal Protein (UniProt) KB database (Bateman 2019), 422 eukaryotic HIF related sequences from 11 gene categories were downloaded in September 2020 (Supplementary Table S1). In July and October 2020, protein sequences from 566 complete eukaryotic genomes were downloaded from the NCBI Reference Sequence database (O’Leary et al., 2016) with assembly levels ranging from *contig* to *complete genome* (Supplementary Table S2). The 566 genomes were classified into 15 phyla according to NCBI taxonomy. The proteins of the 566 eukaryotic genomes were blasted against the eukaryotic HIF related sequence database using DIAMOND v2.0.1.139 (Buchfink et al., 2015), to identify potential HIF related genes. DIAMOND blastp was executed with the options--very-sensitive, --evalue 0.0000001 and--id 25 to define the sensitivity as well as the e-value (10^−7^) and identity (25%) thresholds for the homology search and to determine the best corresponding HIF related genes in each genome. Duplicated sequences for the same identified HIF related gene were removed by only taking one of the best hits.

**FIGURE 2:**
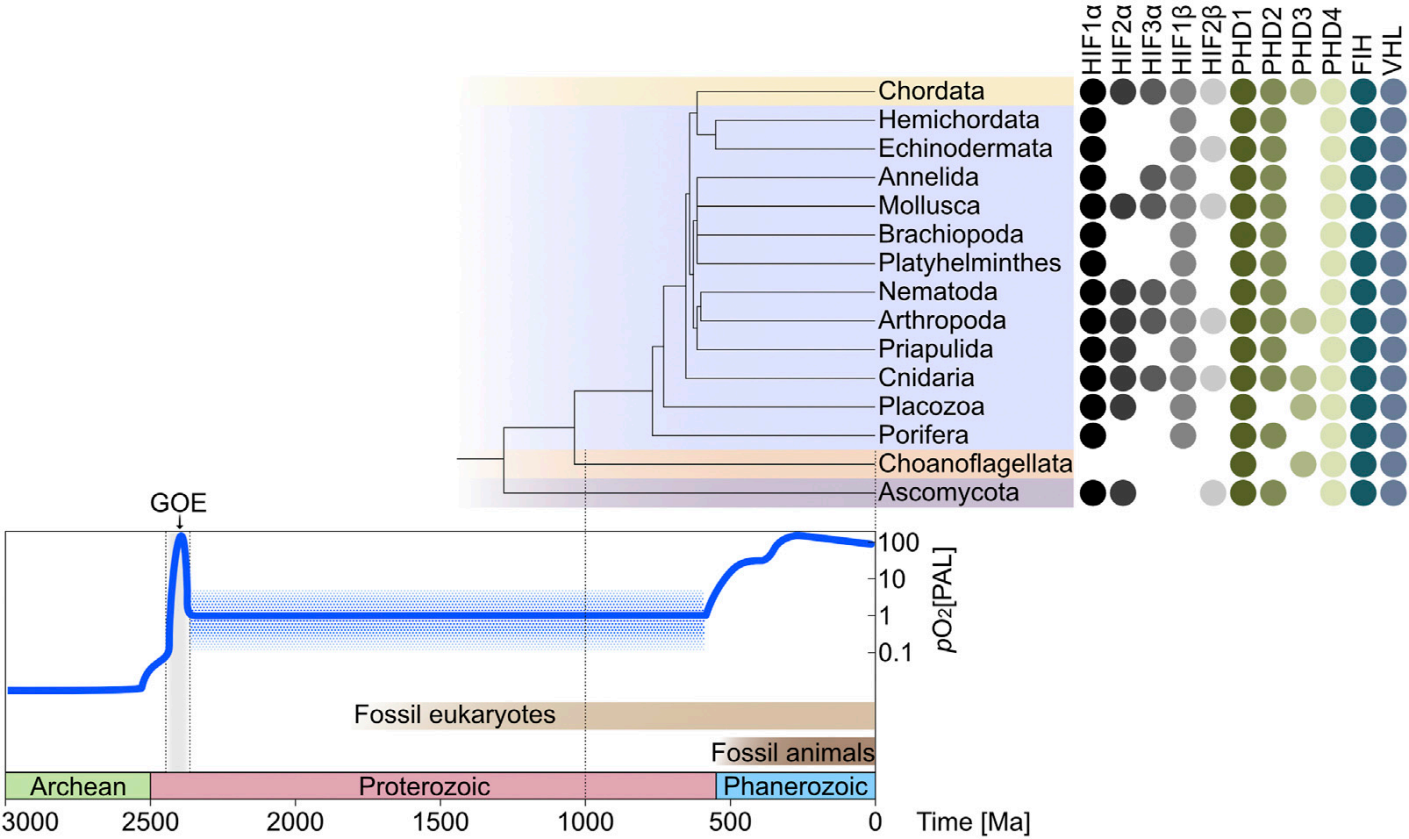
The evolution of the main oxygen sensing pathway across metazoans over the Earth’s oxygen history. The oxygen curve describes the Earth’s oxygen content during each geological time scale (Walker et al., 2018), the detailed information has been obtained from recent publications (Bekker et al., 2004; Lyons et al., 2014). More than 2500 million years (Ma) ago, the Earth oxygen content was almost at an anaerobic state (Catling and Zahnle 2020); during the Proterozoic period (2500–542 Ma), despite the great oxidation event (GOE) that happened at the beginning of this period, the oxygen content was still very low with around 1% present atmospheric levels (PAL) (Allen et al., 2019); during the Phanerozoic period (541–0 Ma), the atmospheric oxygen content increased constantly to the modern level (Lenton et al., 2016; Krause et al., 2018). The phylogenetic tree shown in the figure is scaled to the timeframe at the bottom of the figure. It is connected to the presence-absence pattern (PAP) of oxygen sensing genes. The PAP shows the distribution of the 11 gene categories across 13 metazoan phyla (Chordata, Hemichordata, Echinodermata, Annelida, Mollusca, Brachiopoda, Platyhelminthes, Nematoda, Arthropoda, Priapulida, Cnidaria, Placozoa, and Porifera) and two non-metazoan phyla (Choanoflagellata and Ascomycota) that include 566 species (Supplementary Table S2). The oxygen sensing regulators (HIF1α, HIF2α, HIF3α, HIF1β, and HIF2β) at the left of the PAP are the substrates of the oxygen sensors (PHD1, PHD2, PHD3, PHD4, and FIH) in oxygen-dependent reactions before the ubiquitination (Ub) of the tumor suppressor gene (VHL). (HIF—hypoxia-inducible factor; PHD—HIF prolyl hydroxylases; FIH—factor inhibiting HIF; VHL—von Hippel Lindau tumor suppressor).

Presence and absence patterns of the selected hits were used to demonstrate the evolution of HIF related genes within metazoans over the last 3.0 Ga of Earth’s history (Figure 2). The reference phylogenetic tree in Figure 2 was taken from Telford et al., 2015 and the divergence time of the last common ancestor of each representative phylum on the phylogenetic tree was estimated from the free public database (Kumar et al., 2017) used for the estimation of time-scaled phylogenies (Dos Reis et al., 2015; Gold et al., 2015; Delsuc et al., 2018; Tedersoo et al., 2018). The presence of the genes in each phylum is based on the respective gene being present in at least one species within the indicated phylum and represented by filled colored circles, if the gene was not present in the respective phylum, the space was left blank. The time of the earliest eukaryote fossil record (Eme et al., 2014) is displayed in the time scale at the bottom in light brown and the earliest animal fossil record (Hoyal Cuthill and Han 2018) is indicated by a dark brown bar in the time scale.

### Oxygen affinity of oxygen sensing enzymes and terminal oxidases

K_m_^app^ (O_2_) values of oxygen sensing enzymes (PHD and FIH) and terminal oxidases were collected from literature (Supplementary Tables S3, S4). K_m_ is the Michaelis constant, which represents the concentration of substrate needed to achieve half of the maximum reaction speed (V_max_) of the enzyme (Northrop 1998). K_m_^app^ (O_2_) represents the concentration of oxygen required to achieve half of the V_max_ of the examined enzyme; the lower the K_m_ the higher the affinity of the enzyme for the substrate.

## Results

### The hypoxia induced factor oxygen sensing pathway traces to the last metazoan common ancestor

The two non-metazoan phyla in our dataset (Choanoflagellata and Ascomycota) possess the oxygen sensors PHD, FIH, and VHL. This result is based on the genome of *Salpingoeca rosetta*, as this species deals as a model organism of a group of single-celled eukaryotes that are considered to be the closest relatives of animals (Carr et al., 2008; Booth and King 2022) (Supplementary Table S2). Thus, *S. rosetta* was included to analyze the presence or absence of the HIF-pathway with its components in an organism that may give insight into oxygen sensing *via* hypoxia-inducible factors in simple premetazoan eukaryotes. It has been previously reported that choanoflagellates have PHD, but not HIF and VHL (Rytkönen 2018). Ascomycetes also possess HIF homologs, the result of Ascomycota is based on nine fungi genomes (Supplementary Table S2). Our results support those of Rytkönen et al. in that HIFα and HIFβ subunits are not present in *S. rosetta*, only homologs of PHD1/3/4 as well as FIH and VHL were found. Ascomycota are the largest phylum of fungi predating metazoans, within which Ofd1—the prolyl 4-hydroxylase-like 2-OG-Fe(II) dioxygenase—mediates the degradation and accumulation of the N-terminal transcription factor (Sre1N) (Hughes and Espenshade 2008). Porifera is the sister group of all other animals (Simion et al., 2017) and has been shown to contain HIF1α, HIF1β, PHD1, PHD2, PHD4, FIH, and VHL based on the sponge species *Amphimedon queenslandica* (Supplementary Table S2). *Trichoplax adhaerens* which belongs to the phylum Placozoa is discussed as the last common ancestor of all animals (Schierwater and DeSalle 2018) and was previously shown to possess the key components of the oxygen sensing mechanism—HIFα, PHD, and VHL (Loenarz et al., 2011). Our finding shows a more complete result as *T. adhaerens* not only contains HIFα and PHD homologs but also HIFβ, FIH, and VHL homologues. The main oxygen sensing pathway can thus be traced back to at least the last common ancestor of metazoans, which lived roughly 800 Ma ago, when the atmospheric oxygen content was around 1% PAL (Erwin et al., 2011) (Figure 2).

### The hypoxia induced factor pathway is conserved and widely distributed across metazoans

The 13 metazoan phyla examined all possess HIF1α, HIF1β, PHD1, PHD4, FIH, and VHL. This shows that the core components of the main oxygen sensing pathway are relatively conserved and widespread across metazoans. Moreover, HIF1α is more widely distributed across all phyla and therefore probably evolved earlier than HIF2α and HIF3α (Figure 2). HIF1β is more widely distributed than HIF2β, whereas PHD1 and PHD4 may have arisen earlier than PHD2 and PHD3 as they are found in all phyla examined as shown in Figure 2. It was previously shown that HIF1α is more conserved among metazoans and probably arose before HIF2α (Graham and Presnell 2017), consistent with our findings. However, previous studies have shown that HIF homologs are ubiquitous across metazoans, with the exception of Porifera and Ctenophora, and HIF2α only appears in vertebrates (Loenarz et al., 2011; Rytkönen et al., 2011; Graham and Presnell 2017; Hammarlund et al., 2018; Rytkönen 2018). Accordingly, it has been suggested that the HIF/PHD/FIH/VHL oxygen sensing pathway might represent a recent lineage specific invention among recent animals (Hashimoto et al., 2016; Graham and Barreto 2019; Graham and Barreto 2020). The copepod *Tigriopus californicus* seems to have lost the HIF pathway but still tolerates nearly anoxic conditions for at least 24 h, the genes involved in cuticle reorganization and ion transport may act as the potential solution to low oxygen availability as a replacement to the HIF pathway (Graham and Barreto 2019). *Ramazzottius varieornatus*, one member of the most stress-tolerant tardigrade species, has lost HIF1α, PHD, and VHL selectively (Hashimoto et al., 2016). Three of the four orders of Copepoda were shown to have lost the use of the HIF pathway, but both barnacles and copepods have not fully lost VHL which suggests HIF-independent functions of VHL (Graham and Barreto 2020).

### The oxygen affinity of terminal oxidases is far higher than that of oxygen sensing enzymes

**FIGURE 3:**
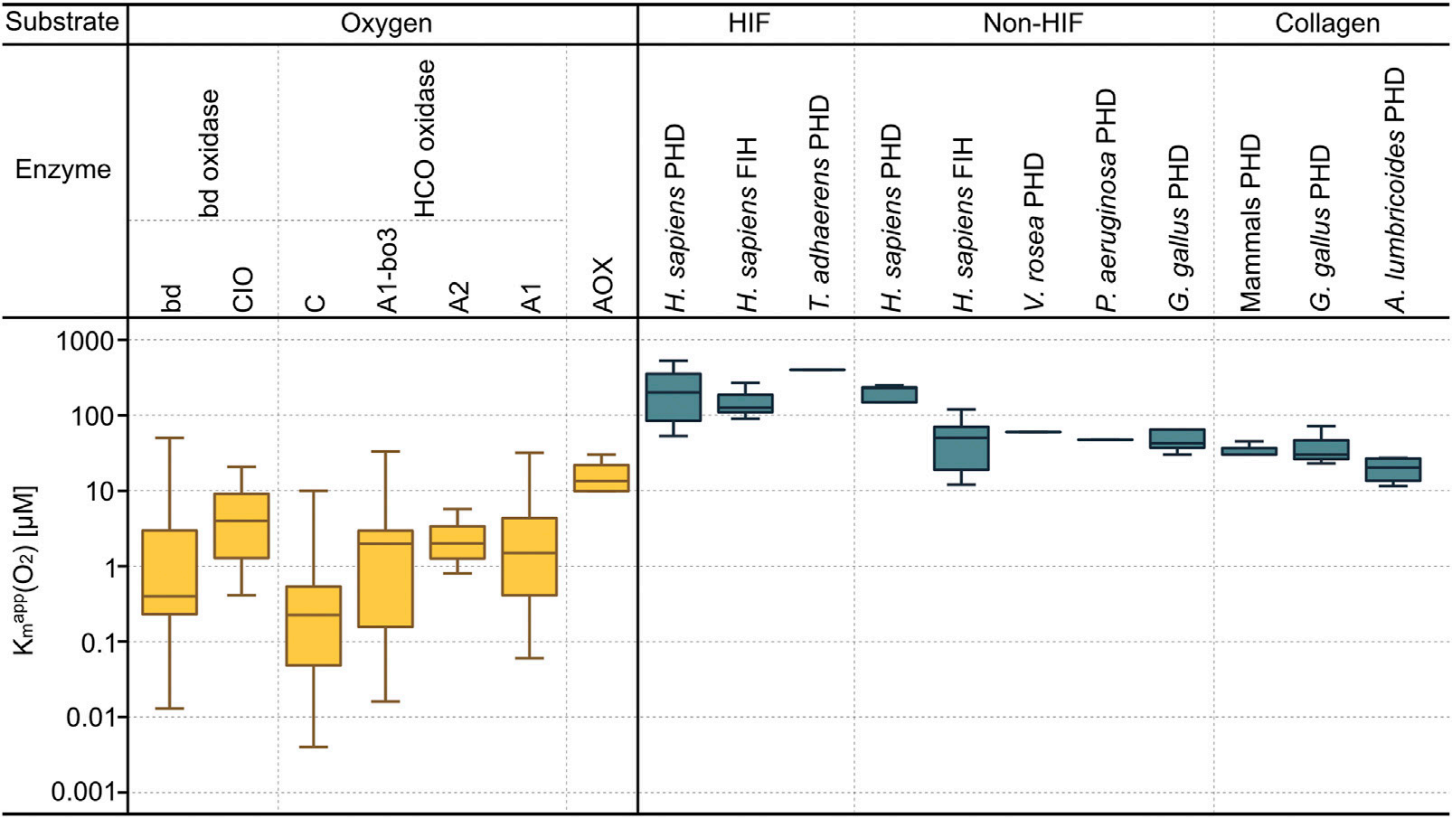
The K_m_^app^(O_2_) values of terminal oxidases and oxygen sensing enzymes. On the left (yellow) K_m_^app^ (O_2_) values of the three main families of terminal oxidases are shown and indicated on the right (dark cyan) are K_m_^app^ (O_2_) values of oxygen sensing enzymes (PHDs and FIH). For cytochrome bd oxidases, two subtypes (bd and CIO) are shown; for HCOs, four subtypes (C, A1-bo3, A2, and A1) are shown. On the top the substrates of the enzymes are indicated; oxygen as substrate for terminal oxidases, as their main function is to reduce oxygen within organism organelles. The K_m_^app^ (O_2_) values of oxygen sensors (PHDs and FIH) are combined based on their substrates, which are sorted into three groups—HIF protein peptides, non-HIF protein peptides, and collagen. All underlying K_m_^app^ (O_2_) values are detailed in Supplementary Tables S3, S4 (AOX: Alternative Oxidase, CIO: Cyanide Insensitive Oxidase, HCO: Heme Copper Oxidase).

The K_m_^app^ (O_2_) of terminal oxidases were obtained from both prokaryotes and eukaryotes, the K_m_^app^ (O_2_) values of oxygen sensing enzymes (PHDs and FIH) were obtained from the literature for eukaryotes (Supplementary Tables S3, S4). Figure 3 shows the distribution of the K_m_^app^ (O_2_) values of terminal oxidases, PHDs and FIH, which are 0.0034–33 μM, 11.54–530 μM and 12–270 μM, respectively. Low K_m_^app^ (O_2_) values indicate higher oxygen affinity, this shows that the oxygen affinity of terminal oxidases is far higher than that of PHD and FIH.

## Discussion

In previous studies, the characteristic domains of HIF, PHD, FIH, and VHL were used to identify HIF related homologs as the characteristic domains are usually the most conserved and representative part of one protein family. Here, complete HIF related protein sequences were used to find possible HIF pathway homologues from sequenced genomes. This identified HIF pathway homologues that were not detected in previous studies. We used Diamond (Buchfink et al., 2015) for searching, which is generally faster and often more accurate than the traditional Basic Local Alignment Search Tool (BLAST; Altschul et al., 1990), which helped to identify previously missing HIF pathway genes.

### The HIF/PHD/FIH/VHL pathway was present during the Cambrian explosion

The largest fungal phylum Ascomycota, the metazoan’s closest sister lineage Choanoflagellata as well as the earliest invertebrate phyla Porifera and Placozoa originated in a low oxygen environment, when the oxygen level was roughly 1%–10% of the present atmospheric level. They are regarded as the low oxygen species group here. According to Figure 2, this group could have the evolved repression machinery - PHD, FIH, and VHL, followed by the first appearance of the HIF1α and HIF1β subunits and gene duplications leading to HIF2α, HIF3α, and HIF2β. The other invertebrate phyla (Cnidaria to Hemichordata in Figure 2) and the vertebrate phylum Chordata appeared before or after the Cambrian period, when the oxygen content increased to the around 10%–100% PAL. Thus, we classify these phyla as the high oxygen group. The HIF oxygen sensing mechanism may have been fully adapted across this group and is responsible for the adaptive transcriptional responses to hypoxia. In this view, the pathway originated in low oxygen environments and evolved vertically in different animal lineages as atmospheric oxygen content rose.

Genes for enzymes of the HIF oxygen sensing pathway—PHD1, PHD4, FIH, and VHL—are distributed across all examined phyla (Figure 2), tracing their first appearance to lineages during the course of metazoan and non-metazoan phylum diversification. HIF1α was the progenitor from which HIF2α and HIF3α arose, and HIF1β may have emerged before HIF2β as it is more widely distributed, giving rise to the hypoxia-inducible transcription factor (HIF1), which is composed of HIF1α and HIF1β, and is the major regulator of oxygen homeostasis (Wang et al., 1995; Semenza 2007). HIFα and HIFβ are only absent in the phylum of Choanoflagellata. As *S. rosetta* is capable of aerotaxis (movement in the direction of oxygen) this species might possess an oxygen sensing mechanism other than HIF because this process requires the ability to detect changes in oxygen levels (Kirkegaard et al., 2016). This alternative oxygen sensing mechanism might also play a role in hypoxia-related responses which obviate a need for retention of HIFα and -β subunits.

Purifying selection is a dominant factor underlying functional diversification and structural conservation among HIFα genes (Brihimi-Horn and Pouysségur 2009; Schödel et al., 2013; Tarade et al., 2019). HIF1α has a high affinity to VHL through the close contact between HIF1α Met561 and VHL Phe91. It is furthermore bound in closer proximity to promoters compared with HIF2α. HIF1β and HIF2β have the same domain architectures and can dimerize with HIF-α subunits, but HIF1β is more conserved across animal lineages than HIF2β. These evolutionary changes accompanied the strict retention of HIF1α and HIF1β across animal lineages.

PHD3 only appears in five of the 13 phyla analyzed but was present in choanoflagellates. Previous work reported that PHD3 may function as an ancient signaling protein due to its involvement in several cell signaling mechanisms (Place and Domann 2013), which enables large animals to sense and deliver oxygen for development and metabolism. Therefore, the retention of PHD3 in choanoflagellates might not reflect the oxygen sensing pathway but other functions instead.

It has been suggested that rudimentary HIF1α-dominant stemness control can function in such a way as to generate oxygen gradients within a tissue in the presence of 1% oxygen and thus maintain the inner tissue’s hypoxic cell stemness, with HIF2α-driven pseudohypoxia maintaining stemness of cells in nearby well-vascularized and oxygenated tissue with more than 1%–3% oxygen (Hammarlund et al., 2018). Under pseudohypoxic conditions, cells can activate hypoxia-related metabolic pathways even though sufficient oxygen supply is provided. Pseudohypoxia conditions can include the accumulation of hypoxia-inducible factor subunits and utilization of glycolysis under normal oxygen conditions in cancer cells (Masoud and Li 2015; Hayashi et al., 2019).

During early animal evolution, refined HIF stemness control might have played an essential role in promoting the larger size of animals and in energy metabolism. Before the Cambrian, low oxygen levels were sufficient to meet the physiological requirements of the simple invertebrates, but after the Cambrian, higher oxygen levels would have interfered with the stemness of animals, which would implicate a role of HIF in the evolution of animal size at the Cambrian explosion. At the same time, collagen hydroxylation at proline residues would have led to more rigid invertebrate body structures and better fossilization properties (Towe 1970).

The functional ranges of both HIF1α and HIF2α (5%–24% PAL or 1%–5% O_2_) hydroxylation (Holmquist-Mengelbier et al., 2006) suggest that they are a relic from the time of O_2_ sensing during low O_2_ concentrations in the Cambrian (15%–20% PAL or 3%–4% O_2_), marking a role for the HIF pathway during the adaptation of larger animals to the rising atmospheric oxygen concentrations. Under low oxygen concentrations of around 1%, both HIF1α and HIF2α are stabilized with HIF1α driving the hypoxia induced reactions which can create oxygen gradients in tissue and thus result in hypoxia and cell stemness within invertebrate tissue. HIF2α may have gained a role for creating the pseudohypoxic phenotype in order to facilitate the activation of genes related with promoting stemness within vertebrate tissue, while HIF2α maintained HIF2α-driven pseudohypoxia, promoting stemness even at oxygen levels of roughly 5% (Hammarlund et al., 2018). Clearly HIF dependent oxygen sensing was integral to the Cambrian explosion (541–485 Ma) and likely facilitated an increase in animal diversity (Knoll and Carroll 1999).

### Low O_2_ affinity of hypoxia induced factor and high O_2_ affinity of terminal oxidases

The K_m_^app^ (O_2_) values in Figure 3 show that the O_2_ affinity of O_2_ sensing enzymes is lower than that of terminal oxidases by one to two orders of magnitude. The O_2_ affinity of A1 type terminal oxidases in mitochondria is on the order of 10 μM, corresponding to 1% [v/v] O_2_ in air reflecting the environmental concentration of O_2_ at the time of mitochondrial origin (Zimorski et al., 2019) which is, in turn, very close to the O_2_ concentration in functioning mitochondria of human tissues because of the oxygen cascade from air to blood to capillaries (Martin et al., 2021). This affinity seems to have changed little during evolution. The situation is different with HIF however, as different animals respond to hypoxia at different levels. For example, *Caenorhabditis elegans* prefers O_2_ levels around 7%, and responds to hypoxia only at about 1% O_2_ (Branicky and Schafer 2008), reflecting diversification of oxygen sensing physiology during metazoan evolution (Kaelin and Ratcliffe 2008; Hampton-Smith and Peet 2009).

PHDs and FIH can target alternative substrates other than HIF, due to their involvement in both HIF and non-HIF related pathways. Their protein hydroxylation is neither unique nor ubiquitous. The substrates of PHDs and FIH can be sorted into three groups (Strowitzki et al., 2019) (Figure 3). Within the oxygen metabolizing enzymes, FIH has a relatively high oxygen affinity compared to the PHDs suggesting that FIH is more hypoxia-tolerant than the PHDs (Tarhonskaya et al., 2015). The K_m_^app^ (O_2_) values of FIH are normally between 90 up to 270 μM (Koivunen et al., 2004; Hangasky et al., 2014; Wilson et al., 2020) while that of PHDs are 30–250 μM (Hirsilä et al., 2003). One study also reported an outlier K_m_^app^ (O_2_) value of PHD around 1700 μM, which is not considered here (Dao et al., 2009). PHD2 was identified as the main regulator of the normal development of growth plate chondrocytes in the avascular environment, as it can inactivate HIF1α to avoid prolonged HIF1α-induced skeletal dysplasia (Stegen et al., 2019). Overexpressed HIF1α can reprogram cellular metabolism from respiration to fermentation (Stegen et al., 2019). Appropriate control of PHD2 on HIF1α activation is also important for collagen synthesis. For the comparison of the same oxygen sensing enzyme type from the same substrate group, it can be seen that larger sized vertebrates have higher oxygen affinity than smaller sized vertebrates and invertebrates on HIF-based substrates and collagen but not non-HIF substrates. This might reflect selection for a greater O_2_ demand for structural rigidity among larger vertebrates that are adapted to life above the soil line.

Within the terminal oxidases, the K_m_^app^ (O_2_) values of other bacteria except for Alphaproteobacteria, archaea, and eukaryotes have also been added here. The mean K_m_^app^ (O_2_) values of these seven of terminal oxidase subtypes, from high to low, are AOX (14.91 μM) > CIO (8.40 μM) > A1-*bo*3 (4.38 μM) > A1 (4.32 μM) > *bd* (4.18 μM) > A2 (2.83 μM) > C (1.62 μM) respectively (Degli Esposti et al., 2019). Cytochrome c oxidase was reported to exhibit a much smaller K_m_^app^ (O_2_) than the mean physiological oxygen concentration compared to other oxygen utilizing enzymes in mammals, while the reaction rates of other oxygen-consuming enzymes could be limited by the physiological oxygen tensions commonly present in organs (Vanderkooi et al., 1991). The lower affinity for O_2_ of HIF dependent O_2_ sensing oxygenases relative to terminal oxidases suggests that the former have undergone evolutionary adaptations in response to changing O_2_ environments that reflect the physiological needs of the whole animal as opposed to its mitochondria, which are supplied *via* circulatory and respiratory systems with roughly the same concentration of O_2_ as the mitochondria of the first unicellular eukaryotes more than 1.5 billion years ago.

## Data Availability

The original contributions presented in the study are included in the article/Supplementary Materials, further inquiries can be directed to the corresponding author.
