# Supplementary material for: The origin and distribution of the main oxygen sensing mechanism across metazoans
Source: Front Physiol. 2022 Oct 17;13:977391. doi: 10.3389/fphys.2022.977391 (PMC9618697; doi:10.3389/fphys.2022.977391)
Supplement: Supplementary file 5 [file DataSheet1.docx]

**Supporting Information**

In this file: References in Supplementary Tables 3 and 4.

References in Supplementary Table 3.

Berchner-Pfannschmidt, U., Tug, S., Trinidad, B., Oehme, F., Yamac, H., Wotzlaw, C. et al. (2008). Nuclear oxygen sensing: induction of endogenous prolyl- hydroxylase 2 activity by hypoxia and nitric oxide. J. Biol. Chem. 283:31745– 31753.

Chrispeels, M. J. (1984). Prolyl hydroxylase in plants. Methods Enzymol. 107:361–369.

Chvapil, M., Boucek, M. & Ehrlich, E. (1970). Differences in the protocollagen hydroxylase activities from Ascaris muscle and hypodermis. Arch. Biochem. Biophys. 140:11–18.

Cianci, P. (2004). Advances in the treatment of the diabetic foot: is there a role for adjunctive hyperbaric oxygen therapy? Wound Repair Regen 12:2–10.

De Jong, L. & Kemp, A. (1984). Stoicheiometry and kinetics of the prolyl 4-hydroxylase partial reaction. ﻿Biochim. Biophys. Acta (BBA)/Protein Struct. Mol. 787:105– 111.

Ehrismann, D., Flashman, E., Genn, D. N., Mathioudakis, N., Hewitson, K. S., Ratcliffe, P. J. et al. (2007). Studies on the activity of the hypoxia-inducible-factor hydroxylases using an oxygen consumption assay. Biochem. J. 401:227–234.

Fujimoto, D. & Prockop, D. J. (1969). Protocollagen proline hydroxylase from Ascaris lumbricoides. J. Biol. Chem. 244:205–210.

Hangasky, J. A., Gandhi, H., Valliere, M. A., Ostrom, N. E. & Knapp, M. J. (2014). The rate-limiting step of O2 activation in the α-ketoglutarate oxygenase factor inhibiting hypoxia inducible factor. Biochemistry 53:8077–8084.

Hirsilä, M., Koivunen, P., Günzler, V., Kivirikko, K. I. & Myllyharju, J. (2003). Characterization of the human prolyl 4-hydroxylases that modify the hypoxia- inducible factor. J. Biol. Chem. 278: 30772–30780.

Hutton, J. J., Tappel, A. L. & Udenfriend, S. (1967). Cofactor and substrate requirements of collagen proline hydroxylase. Arch. Biochem. Biophys. 118:231–240.

Kearney, L. T. 2015. Kinetic investigations of the oxygen entry pathway of the hypoxia- inducible factor (HIF) prolyl hydroxylase 2. Diss. University of Oxford.

Koivunen, P., Hirsilä, M., Günzler, V., Kivirikko, K. I. & Myllyharju, J. (2004). Catalytic properties of the asparaginyl hydroxylase (FIH) in the oxygen sensing pathway are distinct from those of its prolyl 4-hydroxylases. J. Biol. Chem. 279:9899– 9904.

Koivunen, P., Hirsilä, M., Kivirikko, K. I. & Myllyharju, J. (2006). The length of peptide substrates has a marked effect on hydroxylation by the hypoxia-inducible factor prolyl 4-hydroxylases. J. Biol. Chem. 281:28712–28720.

Lippl, K., Boleininger, A., McDonough, M. A., Abboud, M. I., Tarhonskaya, H., Chowdhury, R. et al. (2018). Born to sense: biophysical analyses of the oxygen sensing prolyl hydroxylase from the simplest animal Trichoplax adhaerens. Hypoxia. 6:57.

Liu, T., Abboud, M. I., Chowdhury, R., Tumber, A., Hardy, A. P., Lippl, K. et al. (2020). Biochemical and biophysical analyses of hypoxia sensing prolyl hydroxylases from Dictyostelium discoideum and Toxoplasma gondii. J. Biol. Chem. 295:16545–16561.

Lorenzo, F. R., Huff, C., Myllymäki, M., Olenchock, B., Swierczek, S., Tashi, T. et al. (2014). A genetic mechanism for Tibetan high-altitude adaptation. Nat. Genet. 46:951–956.

Myllyharju, J. 2008. Prolyl 4-hydroxylases, key enzymes in the synthesis of collagens and regulation of the response to hypoxia, and their roles as treatment targets. Ann. Med. 40:402–417.

Myllylä, R., Tuderman, L. & Kivirikko, K. I. (1977). Mechanism of the prolyl hydroxylase reaction: 2. Kinetic analysis of the reaction sequence. Eur. J. Biochem. 80:349– 357.

Pektas, S., Taabazuing, C. Y. & Knapp, M. J. (2015). Increased turnover at limiting O2 concentrations by the Thr387 → Ala variant of HIF-prolyl hydroxylase PHD2. Biochemistry 54:2851–2857.

Scotti, J. S., Leung, I. K., Ge, W., Bentley, M. A., Paps, J., Kramer, H. B. et al. (2014). Human oxygen sensing may have origins in prokaryotic elongation factor Tu prolyl-hydroxylation. PNAS 111:13331–13336.

Tanaka, M., Shibata, H. & Uchida, T. (1980). A new prolyl hydroxylase acting on poly- l-proline, from suspension cultured cells of Vinca rosea. BBA-Enzymol. 616:188–198.

Tarhonskaya, H., Chowdhury, R., Leung, I. K., Loik, N. D., McCullagh, J. S., Claridge, T. D. et al. (2014). Investigating the contribution of the active site environment to the slow reaction of hypoxia-inducible factor prolyl hydroxylase domain 2 with oxygen. Biochem. J. 463:363–372.

Tarhonskaya, H., Hardy, A. P., Howe, E. A., Loik, N. D., Kramer, H. B., McCullagh, J. S. et al. (2015). Kinetic investigations of the role of factor inhibiting hypoxia- inducible factor (FIH) as an oxygen sensor. J. Biol. Chem. 290:19726–19742.

Tryggvason, K., Majamaa, K., Risteli, J. & Kivirikko, K. I. (1979). Partial purification and characterization of chick-embryo prolyl 3-hydroxylase. Biochem. J. 183:303– 307.

Tuckerman, J. R., Zhao, Y., Hewitson, K. S., Tian, Y. M., Pugh, C. W., Ratcliffe, P. J. et al. (2004). Determination and comparison of specific activity of the HIF-prolyl hydroxylases. FEBS Lett. 576:145–150.

Vanderkooi, J. M., Erecinska, M. & Silver, I. A. (1991). Oxygen in mammalian tissue: methods of measurement and affinities of various reactions. Am. J. Physiol. Cell Physiol. 260:1131–1150.

Wilkins, S. E., Hyvärinen, J., Chicher, J., Gorman, J. J., Peet, D. J., Bilton, R. L. & Koivunen, P. (2009). Differences in hydroxylation and binding of Notch and HIF- 1α demonstrate substrate selectivity for factor inhibiting HIF-1 (FIH-1). Int. J. Biochem. Cell Biol. 41:1563–1571.

References in Supplementary Table 4.

Arai, H., Kawakami, T., Osamura, T., Hirai, T., Sakai, Y. & Ishii, M. (2014). Enzymatic characterization and in vivo function of five terminal oxidases in Pseudomonas aeruginosa. J. Bacteriol. 196:4206–4215.

Cooper, C. E. & Brown, G. C. (2008). The inhibition of mitochondrial cytochrome oxidase by the gases carbon monoxide, nitric oxide, hydrogen cyanide and hydrogen sulfide: chemical mechanism and physiological significance. J. Bioenerg. Biomembr. 40:533.

D'Mello, R., Hill, S. & Poole, R. K. (1994). Determination of the oxygen affinities of terminal oxidases in Azotobacter vinelandii using the deoxygenation of oxyleghaemoglobin and oxymyoglobin: cytochrome bd is a low-affinity oxidase. Microbiology 140:1395–1402.

D'Mello, R., Hill, S. & Poole, R. K. (1996). The cytochrome bd quinol oxidase in Escherichia coli has an extremely high oxygen affinity and two oxygen-binding haems: implications for regulation of activity in vivo by oxygen inhibition. Microbiology 142:755–763.

D'Mello, R., Hill, S. & Poole, R. K. (1995). The oxygen affinity of cytochrome bo’ in Escherichia coli determined by the deoxygenation of oxyleghemoglobin and oxymyoglobin: Km values for oxygen are in the submicromolar range. J. Bacteriol. 177:867–870.

Daniel, R. M. (1970). The electron transport system of Acetobacter suboxydans with particular reference to cytochrome o. BBA-Bioenerg. 216:328–341.

Degn, H. & Wohlrab, H. (1971). Measurement of steady-state values of respiration rate and oxidation levels of respiratory pigments at low oxygen tensions. A new technique. BBA-Bioenerg. 245:347–355.

Degli Esposti, M., Mentel, M., Martin, W. & Sousa, F. L. (2019). Oxygen reductases in alphaproteobacterial genomes: physiological evolution from low to high oxygen environments. Front Microbiol 10:499.

Garcia-Horsman, J. A., Barquera, B. & Escamilla, J. E. 1991. Two different aa3‐type cytochromes can be purified from the bacterium Bacillus cereus. Eur. J. Biochem. 199: 761–768.

Gupta, K. J., Zabalza, A. & Van Dongen, J. T. (2009). Regulation of respiration when the oxygen availability changes. Physiol. Plant. 137:383–391.

Hoffman, P. S., Morgan, T. V. & Der Vartanian, D. V. (1979). Respiratory‐chain characteristic of mutants of Azotobacter vinelandii negative to tetramethyl‐p‐ phenylenediamine oxidase. Eur. J. Biochem. 100:19–27.

Ishikawa, R., Ishido, Y., Tachikawa, A., Kawasaki, H., Matsuzawa, H. & Wakagi, T. (2002). Aempyrum pernix K1, a strictly aerobic and hyperthermophilic archaeon, has two terminal oxidases, cytochrome ba3 and cytochrome aa3. Arch. Microbiol. 179:42–49.

Jackson, R. J., Elvers, K. T., Lee, L. J., Gidley, M. D., Wainwright, L. M., Lightfoot, J. et al. (2007). Oxygen reactivity of both respiratory oxidases in Campylobacter jejuni: the cydAB genes encode a cyanide-resistant, low-affinity oxidase that is not of the cytochrome bd type. J. Bacteriol. 189:1604–1615.

Juenemann, S., Butterworth, P. J. & Wrigglesworth, J. M. (1995). A suggested mechanism for the catalytic cycle of cytochrome bd terminal oxidase based on kinetic analysis. Biochemistry 34:14861–14867.

Kita, K., Konishi, K. & Anraku, Y. (1984). Terminal oxidases of Escherichia coli aerobic respiratory chain. J. Biol. Chem. 259:3375–3381.

Kolonay, J. F., Moshiri, F., Gennis, R. B., Kaysser, T. M. & Maier, R. J. (1994). Purification and characterization of the cytochrome bd complex from Azotobacter vinelandii: comparison to the complex from Escherichia coli. J. Bacteriol. 176:4177–4181.

Massari, S., Bösel, A. & Wrigglesworth, J. M. (1996). The variation of Km for oxygen of cytochrome oxidase with turnover under de-energized and energized conditions. Biochem. Soc. Trans. 24:464.

Matsushita, K., Ebisuya, H. & Adachi, O. (1992). Homology in the structure and the prosthetic groups between two different terminal ubiquinol oxidases, cytochrome a1 and cytochrome o, of Acetobacter aceti. J. Biol. Chem. 267:24748–24753.

Miura, H., Mogi, T., Ano, Y., Migita, C. T., Matsutani, M., Yakushi, T. et al. (2013). Cyanide-insensitive quinol oxidase (CIO) from Gluconobacter oxydans is a unique terminal oxidase subfamily of cytochrome bd. J. Biochem. 153:535–545.

Petersen, L. C., Nicholls, P. & Degn, H. (1976). The effect of oxygen concentration on the steady-state kinetics of the solubilized cytochrome c oxidase. BBA- Enzymol. 452:59-65.

Pils, D. & Schmetterer, G. (2001). Characterization of three bioenergetically active respiratory terminal oxidases in the cyanobacterium Synechocystis sp. strain PCC 6803. FEMS Microbiol. Lett. 203:217–222.

Preisig, O., Zufferey, R., Thöny-Meyer, L., Appleby, C. A. & Hennecke, H. (1996). A high-affinity cbb3-type cytochrome oxidase terminates the symbiosis-specific respiratory chain of Bradyrhizobium japonicum. J. Bacteriol. 178:1532–1538.

Ribas-Carbo, M., Berry, J. A., Azcon-Bieto, J. & Siedow, J. N. (1994). The reaction of the plant mitochondrial cyanide-resistant alternative oxidase with oxygen. BBA- Bioenerg. 1188:205–212.

Rice, C. W. & Hempfling, W. P. (1978). Oxygen limited continuous culture and respiratory energy conservation in Escherichia coli. J. Bacteriol. 134:115–124.

Smith, A., Hill, S. & Anthony, C. (1990). The purification, characterization and role of the d-type cytochrome oxidase of Klebsiella pneumoniae during nitrogen fixation. J. Gen. Microbiol. 136:171–180.

Sone, N., & Fujiwara, Y. (1991). Haem O can replace haem A in the active site of cytochrome c oxidase from thermophilic bacterium PS3. FEBS Lett. 288:154– 158.

Sone, N., Kutoh, E. & Sato, K. (1990). A cytochrome o-type oxidase of the thermophilic bacterium PS3 grown under air-limited conditions. J. Biochem. 107:597–602.

Vanderkooi, J. M., Erecinska, M. & Silver, I. A. (1991). Oxygen in mammalian tissue: methods of measurement and affinities of various reactions. Am. J. Physiol. Cell Physiol. 260:1131-1150.

Wilson, D. F., Erecinska, M., Drown, C. & Silver, I. A. (1977). Effect of oxygen tension on cellular energetics. Am. J. Physiol. Cell Physiol. 233:135–140.

Yamanaka, T., Ota, A. & Okunuki, K. (1961). A nitrite reducing system reconstructed with purified cytochrome components of Pseudomonas aeruginosa. Biochim. Biophys. Acta 53:294–308.

Zimorski, V., Mentel, M., Tielens, A. G. & Martin, W. F. (2019). Energy metabolism in anaerobic eukaryotes and Earth’s late oxygenation. Free Radic. Biol. Med 140:279–294.
